# National safety monitoring of vaccines from the Canadian Adverse Events Following Immunization Surveillance System (CAEFISS), 2018–2019

**DOI:** 10.14745/ccdr.v50i12a06

**Published:** 2024-01-01

**Authors:** Maryem El Jaouhari, Karin Johnson, Helen Anyoti, Yuhui Xu, Charlotte Wells, Ashley Weeks, Allison Yeung, Amanda Shaw, Susanna Ogunnaike-Cooke

**Affiliations:** 1 Centre for Immunization Surveillance, Public Health Agency of Canada, Ottawa, ON

**Keywords:** vaccine safety, pharmacovigilance, adverse events following immunization

## Abstract

**Background:**

The Canadian Adverse Events Following Immunization Surveillance System (CAEFISS) is a comprehensive vaccine safety surveillance system that includes both passive and active surveillance of vaccines administered in Canada. This work presents a summary of adverse events following immunization (AEFI) nationally for 2018 and 2019.

**Methods:**

Data extracted from CAEFISS included all AEFI reports received by the Public Health Agency of Canada by April 30, 2022, for vaccines marketed in Canada and administered between January 1, 2018, and December 31, 2019. Descriptive statistics were conducted on AEFI reports by type of surveillance program (i.e., active vs. passive), AEFIs, demographics, healthcare utilization, outcome, seriousness of adverse events and type of vaccine.

**Results:**

Between 2018 and 2019, 5,875 AEFI reports were received from across Canada. The average annual AEFI reporting rate was 10.9/100,000 doses distributed in Canada for vaccines administered during 2018–2019 and was found to be inversely proportional to age. The majority of reports (91%) were non-serious events, involving vaccination site reactions, rash and allergic events. Overall, there were 511 serious adverse event reports during 2018–2019. Of the serious adverse event reports, the most common primary AEFIs were anaphylaxis followed by seizure. There were no unexpected vaccine safety issues identified or increases in frequency or severity of adverse events.

**Conclusion:**

Canada’s continuous monitoring of the safety of marketed vaccines during 2018–2019 did not identify any increase in the frequency or severity of AEFIs, previously unknown AEFIs, or areas that required further investigation or research.

## Introduction

Vaccine safety surveillance is essential to detect any emerging issues or changes in the frequency of adverse events following immunization (AEFI). The Public Health Agency of Canada (PHAC) and Health Canada share the monitoring of the safety of vaccines in Canada.

The Canadian Adverse Events Following Immunization Surveillance System (CAEFISS) is a federal, provincial, and territorial (FPT) public health post-market vaccine safety surveillance system ((1)). The CAEFISS is managed by PHAC and is unique in that it includes both passive (spontaneous reports from FPTs) and active surveillance. The primary objectives of CAEFISS are to 1) continuously monitor the safety of marketed vaccines in Canada, 2) identify increases in the frequency or severity of previously identified vaccine-related reactions, 3) identify previously unknown AEFIs that could possibly be related to a vaccine, 4) identify areas that require further investigation and/or research and 5) provide timely information on AEFI reporting profiles for vaccines marketed in Canada, which could help inform immunization programs and guidelines ((1)).

In Canada, healthcare providers, manufacturers and the public each have a role to play in vaccine pharmacovigilance ((2)). Federal, provincial and territorial public health officials monitor vaccine safety through the Vaccine Vigilance Working Group (VVWG) of the Canadian Immunization Committee (CIC). The VVWG includes representatives from all FPT immunization programs across the country as well as Health Canada regulators and the Canadian Immunization Monitoring Program ACTive (IMPACT) surveillance system.

For more information about CAEFISS, IMPACT and VVWG, please refer to the **Technical Annex**, **Supplemental material**, for annual vaccine safety reports. In addition, a more comprehensive description of the roles and responsibilities for post-market pharmacovigilance can be found in the Canadian Immunization Guide and on the CAEFISS webpage ((1,2)). Details on provincial and territorial vaccination schedules can be found on the PHAC website ((3)). National reports on vaccine safety surveillance data have been published periodically using CAEFISS data ((4–15)).

The objectives of this report are to provide 1) a descriptive analysis of AEFI reports submitted to CAEFISS for vaccines administered in Canada in 2018–2019, 2) a descriptive review of healthcare utilization and outcome following an AEFI and 3) an analysis of serious adverse events (SAEs).

## Methods

### Definitions

An AEFI is defined as any untoward medical occurrence that follows immunization but that does not necessarily have a causal relationship with the administration of the vaccine. The adverse event may be a sign, symptom or defined illness ((15)).

A serious AEFI in CAEFISS is identified based on International Council for Harmonisation of Technical Requirements for Pharmaceuticals for Human Use as an event that results in death, is life-threatening, and requires inpatient hospitalization or prolongation of existing hospitalization, results in persistent or significant disability/incapacity or results in a congenital anomaly/birth defect. Any medical event which may not be immediately life-threatening but requires intervention to prevent one of the outcomes listed above may also be considered as serious ((16)).

### Data sources

The CAEFISS combines reports from passive and active surveillance. Active surveillance is conducted by IMPACT, a network of 12 tertiary care paediatric hospitals across Canada that screens hospital admissions for specific AEFIs. Passive surveillance is initiated at the local public health level and relies on reporting of AEFIs by healthcare providers, vaccine recipients or their caregivers.

We searched CAEFISS for all AEFI reports received by April 30, 2022, with a date of vaccine administration between January 1, 2018, and December 31, 2019. The AEFI report forms used in Canada collect information on sex, age, vaccine administered, medical history, concomitant medications and adverse events experienced. In addition, historic AEFI reports with a date of vaccine administration between 2008 and 2017 were extracted from CAEFISS to assess trends over time. It should be noted that for one province/territory, not all AEFI reports were included due to technical issues with uploading the information onto the CAEFISS platform. The reports that were not included from this province/territory were of a small enough volume that this issue did not impact our confidence in the results.

### Data analysis

Descriptive analyses were conducted for AEFI reports by year, type of surveillance (active vs. passive), primary reason for reporting, seriousness, healthcare utilization and outcome. Rates of AEFI were calculated using dose distributed data where possible. Sex and age-specific AEFI rates were calculated using population estimates as the denominator. Missing data were excluded from the calculations. All reports were medically reviewed and only reports with an outcome of death underwent causality assessment for this report. All analyses were conducted using SAS EG 7.1 and Microsoft Excel 2016.

### Technical annex

The Technical Annex contains detailed methodological descriptions of the AEFI reporting process as well as the data extraction and analysis of the surveillance data. In addition, the annex includes information on AEFI surveillance definitions (i.e., primary AEFI, active and passive surveillance), how rates are reported, limitations of CAEFISS, information regarding vaccine abbreviations and marketed product/trade names, medical case review AEFI categories/subcategories and information on the severity classification for primary AEFIs in the medical case review.

## Results

**Figure 1:**
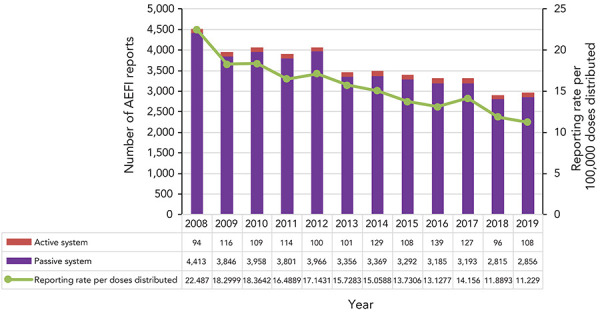
Total number of adverse events following immunization reports and reporting rate by reporting source, 2008–2019 (N=5,875)^a^ Abbreviation: AEFI, adverse event following immunization ^a^ Does not include the influenza A(H1N1) 2009 pandemic influenza AEFI reports

Our search identified a total of 5,875 AEFI reports between 2018 and 2019 in CAEFISS from 12 provinces and territories: 2,911 AEFI reports from 2018 and 2,964 AEFI reports from 2019. During this period, over 50 million vaccine doses were distributed. This represented an AEFI reporting rate of 11.5 per 100,000 doses distributed in 2018 and 2019. Over the preceding 11 years, the AEFI reporting rate decreased from 22.5 in 2008 to 11.2 per 100,000 doses distributed in 2019 (*p*<0.01) (**Figure 1**). The reduction in reporting occurred from the passive surveillance source, while the annual number of reports from active surveillance remained generally stable over time.

### Distribution of adverse events following immunization by age group

**Figure 2:**
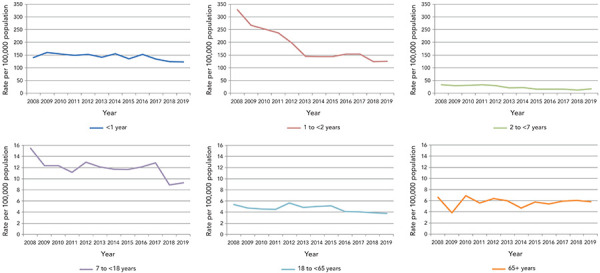
Annual rate of adverse events following immunization by age group, 2008–2019 (N=5,814)^a^ ^a^ 61 reports with missing age and sex are excluded

The median age of all cases reported in 2018–2019 at time of vaccination was 12 years (range: newborn to 100 years). The majority (55%) of AEFI reports were for children and adolescents under 18 years of age. Age-specific reporting rates were higher for younger age groups and lower for older age groups. Rates were highest for children younger than one year of age (123.9 per 100,000 population), followed by children one to younger than two years of age (123.6 per 100,000 population) (**Figure 2**). Among other age groups, the reporting rates were lower than 50 per 100,000 population.

Overall, between 2008 and 2019, decreases in AEFI reporting rates were seen in all age groups. The largest decrease (−62%) was seen in the 1 to <2 years age group, followed by the 2 to <7 years age group (−50%). The rate in the latter age group had slightly increased from 2018 to 2019 (+40%).

### Distribution of adverse events following immunization by sex

**Figure 3:**
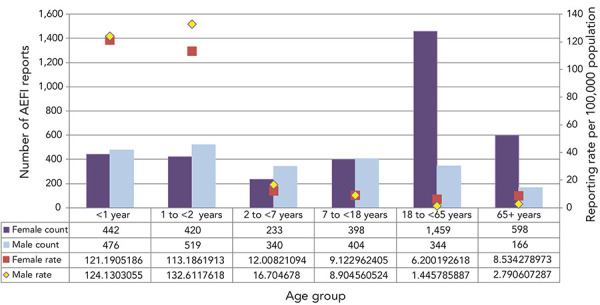
Number and reporting rate of adverse events following immunization reports by age group and sex, 2018–2019 (N=5,814)^a^ Abbreviation: AEFI, adverse events following immunization ^a^ 61 reports with missing age and sex were excluded

Of the 5,875 reports, 60.5% were for females. As shown in **Figure 3**, there appears to be a slight male predominance of adverse events observed for children younger than seven years of age. Most age groups had relatively similar rates for males and females; however, within the age groups 18–64 years and 65 years and older the rate was higher for females.

### Primary reason for reporting adverse events following immunization

**Figure 4:**
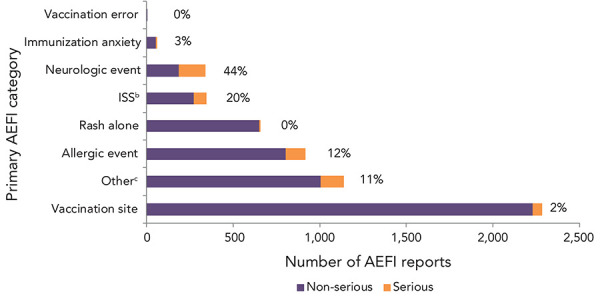
Primary adverse events following immunization category by seriousness, 2018–2019 (N=5,726)^a,b,c^ Abbreviations: AEFI, adverse event following immunization; ISS, infection/syndrome/systemic symptoms ^a^ 149 reports with missing primary AEFI are excluded ^b^ Infection/syndrome/systemic symptoms are primarily events involving many body systems often accompanied by fever. They include sub-categories such as recognized syndromes (e.g., Kawasaki syndrome, fibromyalgia, etc.), fever alone, influenza-like illness, and systemic events (such as fatigue, malaise and lethargy). They also include symptoms occurring in one or more body parts ^c^ Other includes arthralgia, arthritis, hypotonic-hyporesponsive episode, intussusception, gastrointestinal disorder, parotitis, persistent crying, rash and thrombocytopenia

**Table 1** provides a breakdown of reasons for submitting an AEFI report. The percentage of serious reports for each AEFI subcategory and the overall category is shown in the table. Vaccination site reactions were the most common primary AEFI reported (n=2,283, 40%) followed by allergic or allergic-like events (n=915, 16%) and rashes (n=653, 11%). These three categories combined represent 67% of all adverse event reports and 14% of all SAE reports submitted in 2018–2019. **Figure 4** shows that the proportion of serious events was highest for the neurological event category (44%), followed by infection/syndrome/systemic symptoms (20%). Of note, only one report included vaccination errors, which was not a serious event. All AEFI reports were medically reviewed.

**Table 1:** Frequency of reports and percent that is serious for each primary adverse event following immunization subcategory, 2018–2019 (N=5,726)

Main reason for reporting	Detailed reason for reporting^a^	Number of reports	Serious reports (%)
Allergic or allergic-like events	Events managed as anaphylaxis	101	100
	Oculo-respiratory syndrome	61	0
	Other allergic events^b^	753	1
	Total	915	12
Infection/syndrome/systemic symptoms^c^	Fever only	22	9
	Influenza-like illness	13	0
	Infection	79	27
	Rash with fever and/or other illness	115	4
	Syndromes	39	82
	Systemic	75	12
	Total	343	20
Immunization anxiety	Other anxiety-related^d^	9	0
	Presyncope	12	8
	Syncope	37	3
	Total	58	3
Neurologic events	Aseptic meningitis	5	100
	Ataxia/cerebellitis^e^	3	67
	Bell’s palsy	14	14
	Encephalitis/acute disseminated encephalomyelitis/myelitis	14	100
	Guillain-Barré syndrome	13	92
	Other neurologic event^f^	64	30
	Seizure	223	43
	Total	336	44
Other	Arthralgia	34	6
	Arthritis	20	10
	Gastrointestinal event	452	7
	Hypotonic-hyporesponsive episode	48	31
	Intussusception	16	75
	Other events^g^	427	9
	Paraesthesia/anaesthesia	61	2
	Parotitis	8	0
	Persistent crying	37	0
	Sudden unexpected/unexplained death syndrome	2	100
	Thrombocytopenia	30	90
	Vaccination failure	1	100
	Total	1,136	11
Rash alone	Extent unknown	36	0
	Generalized	530	0
	Localized	87	0
	Total	653	0
Vaccination error	Total	1	0
Vaccination site reactions	Abscess	21	19
	Cellulitis	649	4
	Extensive limb swelling^h^	187	2
	Other local reaction^i^	1,291	1
	Limb pain more than 7 days	135	1
	Total	2,283	2

^a^ 149 reports with missing primary adverse events following immunization (AEFI) category

^b^ Other includes, but is not limited to, hypersensitivity and urticarial

^c^ Infection/syndrome/systemic symptoms are primarily events involving many body systems often accompanied by fever. They include sub-categories such as recognized syndromes (e.g., Kawasaki syndrome, fibromyalgia, etc.), fever alone, influenza-like illness and systemic events (such as fatigue, malaise and lethargy). They also include symptoms occurring in one or more body parts

^d^ Other includes, but is not limited to, dizziness and dyspnoea

^e^ Cerebellar ataxia is defined as sudden onset of truncal ataxia and gait disturbances ((17)). Of note, this assumes absence of cerebellar signs appearing with other evidence of encephalitis or Acute Disseminated Encephalomyelitis (ADEM), in which case it would be classified according to the Brighton-Collaboration case definition ((18))

^f^ Other includes, but is not limited to, seizure-like phenomena and migraine

^g^ Other includes, but is not limited to, lymphadenopathy and arthralgia

^h^ Extensive limb swelling of an entire proximal and/or distal limb segment with segment defined as extending from one joint to the next ((19))

^i^ Other includes, but is not limited to, vaccination site pain and vaccination site swelling

**Figure 5:**
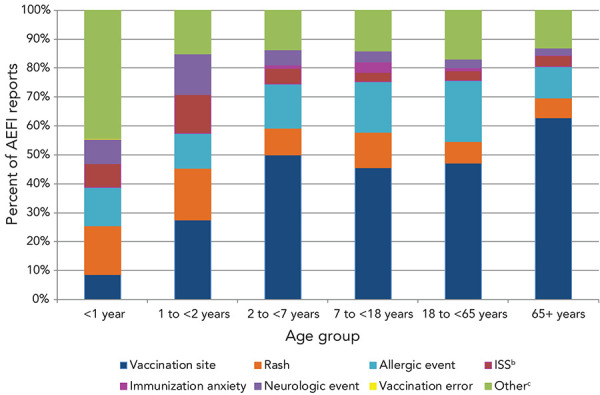
Distribution of primary adverse events following immunization reported by age group, 2018–2019 (N=5,726)^a,b,c^ Abbreviations: AEFI, adverse event following immunization; ISS, infection/syndrome/systemic symptoms ^a^ 149 reports with missing age and three reports with missing primary AEFI are excluded ^b^ ISS are primarily events involving many body systems often accompanied by fever. They include sub-categories such as recognized syndromes (e.g., Kawasaki syndrome, fibromyalgia, etc.), fever alone, influenza-like illness, and systemic events (such as fatigue, malaise, and lethargy). They also include symptoms occurring in one or more body parts ^c^ Other includes arthralgia, arthritis, hypotonic-hyporesponsive episode, intussusception, gastrointestinal diseases, parotitis, persistent crying, rash and thrombocytopenia

**Figure 5** shows the distribution of AEFI by primary reason for reporting by age group. Vaccination site reactions represented the greatest number of AEFIs for all age groups except for children younger than one year of age. For children under the age of one, excluding the “other” event category, the most commonly reported AEFI was rash, followed by allergic event.

### Serious adverse event reports

**Figure 6:**
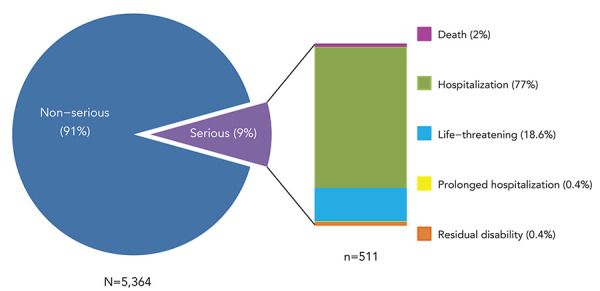
Classification of serious adverse event reports, 2018–2019^a^ ^a^ Percentages in figure do not total 100% due to rounding

There were 511 SAE reports out of over 50 million vaccine doses distributed during the reporting period. This represents a reporting rate of 0.7/100,000 doses distributed and 9% of all AEFI reports for the 2018–2019 period. **Figure 6** shows the distribution of SAE reports by reason for seriousness, with hospitalization (77%) and life-threatening events (19%) being the most common.

The most frequently cited reason for reporting was events managed as anaphylaxis (n=101, 20%), followed by seizure (n=95, 18.6%). The majority (n=364, 71%) of SAE reports had fully recovered at the time of reporting. For those patients who had not fully recovered at the time of reporting, these reports were revised if updated information was received by CAEFISS from the reporting federal, provincial or territorial health authority. Other outcomes for SAE reports included fatal outcome (n=10, 2%) and permanent disability/incapacity (n=2, 0.4%). The majority of SAE reports were for children and adolescents younger than 18 years of age (n=397, 78%), with three quarters (75%) of these reported for children younger than two years of age.

All 10 reports of death underwent careful medical review, and none was considered to be related to the vaccines administered. There were seven deaths in children younger than five years of age; three of the deaths were considered to be a result of pre-existing medical conditions and another three were due to infections unrelated to vaccination. The seventh case had insufficient information, with the cause of death reported as unknown. Three deaths were reported in adults older than 70 years of age and were as a result of pre-existing medical conditions. There were two additional cases where outcome of persistent/significant disability were present at the time of reporting. No longer term outcome information was obtained on these cases.

### Healthcare utilization

**Table 2** shows the reported highest level of care sought following an AEFI. The most frequently reported was a non-urgent healthcare visit (43%). Most people with a reported AEFI (91%) did not require hospitalization. In 24% of the reports, no health care was sought, and these may have included self-reporting of milder AEFIs to public health units or healthcare providers.

**Table 2:** Highest level of health care sought for adverse events following immunization, 2018–2019 (N=5,489)

Highest level of care sought (N=5,489ᵃ)	n	%ᵇ
Non-urgent visit	2,369	43
None	1,319	24
Emergency visit	1,119	20
Required hospitalization	405	7
Telephone advice from a health professional	195	4
Resulted in prolongation of existing hospitalization	1	0
Vaccination clinic	1	0
Unknown^c^	80	1

^a^ The 386 reports with missing information on highest level of care sought were excluded. There is variation across provinces and territories with respect to data collection on levels of health care sought. Data is not collected at all levels by all provinces and territories

^b^ Percentages in table do not total 100% due to rounding

^c^ Unknown is selected only when the level of care sought is indicated as unknown in the report

### Outcome

The outcome at time of reporting for all AEFI reports is shown in **Table 3**. Full recovery was indicated for 74% of the reports and 0.2% of reports reported death as an outcome.

**Table 3:** Outcome at time of reporting for all adverse events following immunization reports, 2018–2019 (N=5,753)^a^

Outcome	Number of reports	Proportion of reports%ᵇ
Fully recovered	4,244	74
Not yet recovered	1,222	21
Unknown^c^	275	5
Permanent disability/incapacity	2	0.0
Death	10	0.2

^a^ 122 cases were missing information on outcome, therefore were excluded

^b^ Percentages in table do not total 100% due to rounding

^c^ Unknown is selected only when the outcome is indicated as unknown in the report

## Discussion

In 2018–2019, the overall AEFI reporting rate was 11.5 per 100,000 doses distributed and represented a statistically significant decrease in reporting rates over the preceding 11 years. The decline in reporting rates over time may be due to increasing familiarity with expected side effects from vaccine over time (associated with reduced health care seeking for adverse events), under-reporting and changes in reporting requirements by jurisdictions over time. In comparison to the 2018 and 2019 Australian annual reporting rate of 16.9 and 14.9 per 100,000 population, respectively, the Canadian reporting rate is lower, which may in part be due to differences in case definitions used, immunization schedules, AEFI surveillance systems, reporting practices and population demographics ((20,21)). No vaccine safety issues or increases in frequency or severity of adverse events were identified by VVWG during the reporting period.

The majority of AEFI reports involved vaccines given to infants and young children younger than two years of age. This was expected, given that this age group receives many vaccines, both at a single visit and spaced closer together, leading to more opportunities for adverse events to be temporally associated with immunization and reported to a healthcare provider. For all age groups, a significant decrease in AEFI reporting occurred over the preceding 11 years, with the greatest decrease seen in the one to younger than two years old age group. A greater proportion of reports involved females; similar to other findings where female adults were found to consistently report more adverse events ((6,13,14,22,23)). The reported sex differences in AEFI counts and rates by age may also be explained in part by higher vaccine coverage in female adults ((24)). This is similar to other studies of sex-specific differences in AEFI reporting rates ((20–23,25)).

The majority of reported adverse events from approximately 50 million doses of vaccine distributed in Canada were non-serious vaccination site reactions, such as pain and redness, and allergic events, such as hypersensitivity and rash. In 2018–2019, 9% of AEFIs reported were SAEs. Comparing this to other countries that use the same definition for a SAE, this proportion is higher compared to that reported in New Zealand (3.5%) for the same time period ((26)) and is lower than that reported in Australia in 2018 and 2019 (16% and 12%) ((20,21)). It is also similar to previous years in Canada (8% and 9%) ((6,14)). The variations in proportions seen among different countries may be due in part to differences in methodology as stated previously.

The majority of reported SAEs occurred in children and adolescents, which may in part be explained by IMPACT, which actively searches for specific surveillance targets in children admitted to 12 paediatric tertiary care hospitals in Canada, resulting in a higher reporting rate in this age group ((27–29)). Immunization Monitoring Program ACTive contributed 6% of all AEFI reports and 50% of all serious reports in children under the age of 18 years, which is similar to the results reported in the 2013–2016 and 2017 reports for this age group. In all age categories, the proportion of SAEs was highest for the neurological event category, for which IMPACT specifically searches. No discernable trends were identified for the number of specific serious adverse events reported over the 2008–2019 time period. Regarding the most frequently cited reason for reporting among SAE reports for all age groups combined, the number and rate of reports of seizures have decreased from 0.45 to 0.19 per 100,000 doses distributed since 2016 and is below the expected frequency (very rare: less than 1/10,000 doses distributed) identified by the World Health Organization ((30)). The number and rate of reports of events managed as anaphylaxis have remained relatively stable since 2016 with an annual reporting rate of 0.20 per 100,000 doses distributed (two per million doses distributed), which is within the expected range of one to 10 episodes per million doses of vaccines administered ((31)). For both seizure reports and reports for events managed as anaphylaxis, the reports were distributed across multiple ages and vaccines with no lot-specific clusters. It should be noted that case definitions for events managed as anaphylaxis vary slightly by province and territory. In general, the definition of anaphylaxis is intentionally very sensitive to ensure that all potential cases of anaphylaxis are captured. At the time of reporting, the majority of individuals with SAEs had fully recovered. Of the 10 deaths reported over the two-year period, none were found to be related to the vaccines administered.

## Limitations

Passive surveillance for AEFIs is subject to limitations such as under-reporting, lack of certainty regarding the diagnostic validity of a reported event, missing information regarding other potential causes such as pre-existing medical conditions or concomitant medications and differing AEFI reporting practices by jurisdictions within Canada. Passive surveillance detects temporal events; however, from the AEFIs described in this paper, causal inferences cannot be made since causality assessment was only conducted for reports that stated an outcome of death. Despite these limitations, passive surveillance is an essential tool for detecting potential vaccine safety signals, especially new or unusual adverse events too rare to assess during clinical trials. Seasonality was not analyzed as a potential variable in this report.

There are also limitations associated with active surveillance. Immunization Monitoring Program ACTive uses predetermined AEFI targets (such as seizure), which may limit its ability to identify new adverse reactions to immunizations. In addition, while IMPACT covers 90% of Canada’s tertiary care pediatric beds and hospital admissions, its focus is on admitted paediatric cases, which means only the most serious cases are detected ((29,30)).

The number of doses administered in the population is not available at the national level; therefore, the denominator used in rate calculations was either doses distributed or population statistics. The use of doses distributed as the denominator can underestimate rates, as they do not take unused doses or wastage into account. Furthermore, doses distributed in one year may not be administered in that same year, further limiting the accuracy of the doses distributed denominator. Despite these limitations, a doses distributed-based denominator for rate calculations was used when possible in this report, as a population-based denominator assumes similar distribution of vaccine doses across population subgroups, and this may not be true in all cases.

## Conclusion

There were no vaccine safety issues identified or increases in frequency or severity of expected adverse events in the CAEFISS data. The majority of reported AEFIs were expected and mild in nature and the overall proportion of serious adverse events were similar to previous years.

## Supplemental material

These documents can be accessed on the Supplemental material file.
